# Supplementary material for: Ectoparasites enhance survival by suppressing host exploration and limiting dispersal
Source: Nat Commun. 2025 May 9;16:4318. doi: 10.1038/s41467-025-59601-9 (PMC12064801; doi:10.1038/s41467-025-59601-9)
Supplement: Supplementary file 1 — Supplementary Information [file 41467_2025_59601_MOESM1_ESM.pdf]

Ectoparasites Enhance Survival by Suppressing Host Exploration and Limiting Dispersal

Liu *et.al*

Supplementary Information

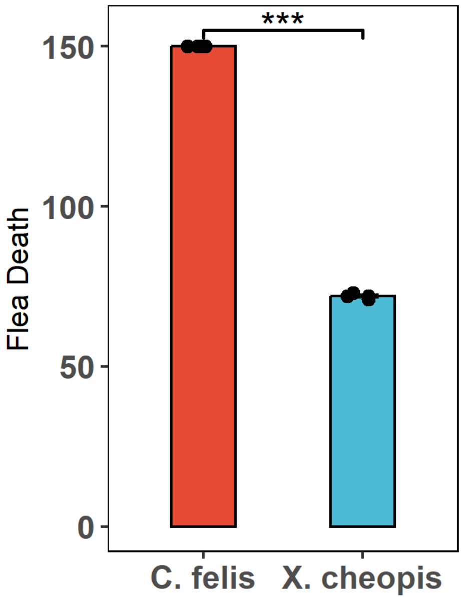

Supplementary Fig 1. Number of dead fleas per cage one week after infection. Red represents *Ctenocephalides felis* (n=4), and blue represents *Xenopsylla cheopis* (n=4). One-sided t test was used. Each black dot represents one cage. Data are presented as mean ± SEM. Source data are provided as a Source Data file. ***$p < 0.001$

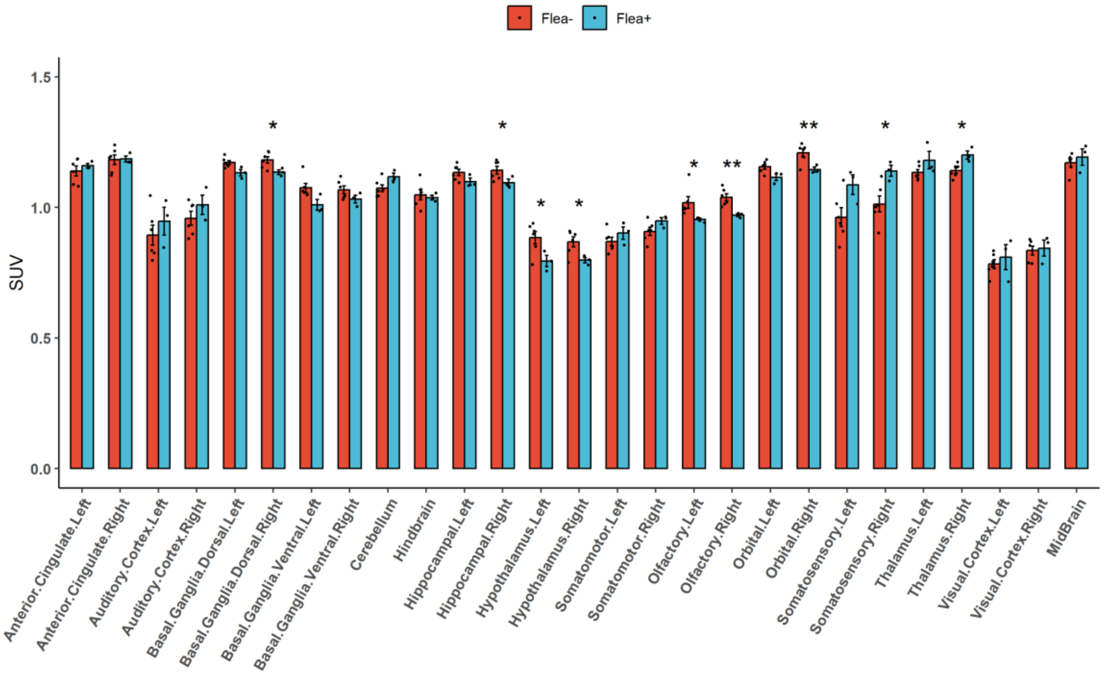

Supplementary Fig 2. The standard uptake value (SUV) for ^{18}F-FDG in each brain region based on PET-CT for Flea− (n=6) and Flea+ (n=3) groups. Two-sided t test was used. Red represents the Flea− group, and blue represents the Flea+ group. Each black dot represents an individual. Asterisks indicates brain regions with significant differences. Data are presented as mean ± SEM. Source data are provided as a Source

Data file. $**p < 0.01$, $*p < 0.05$

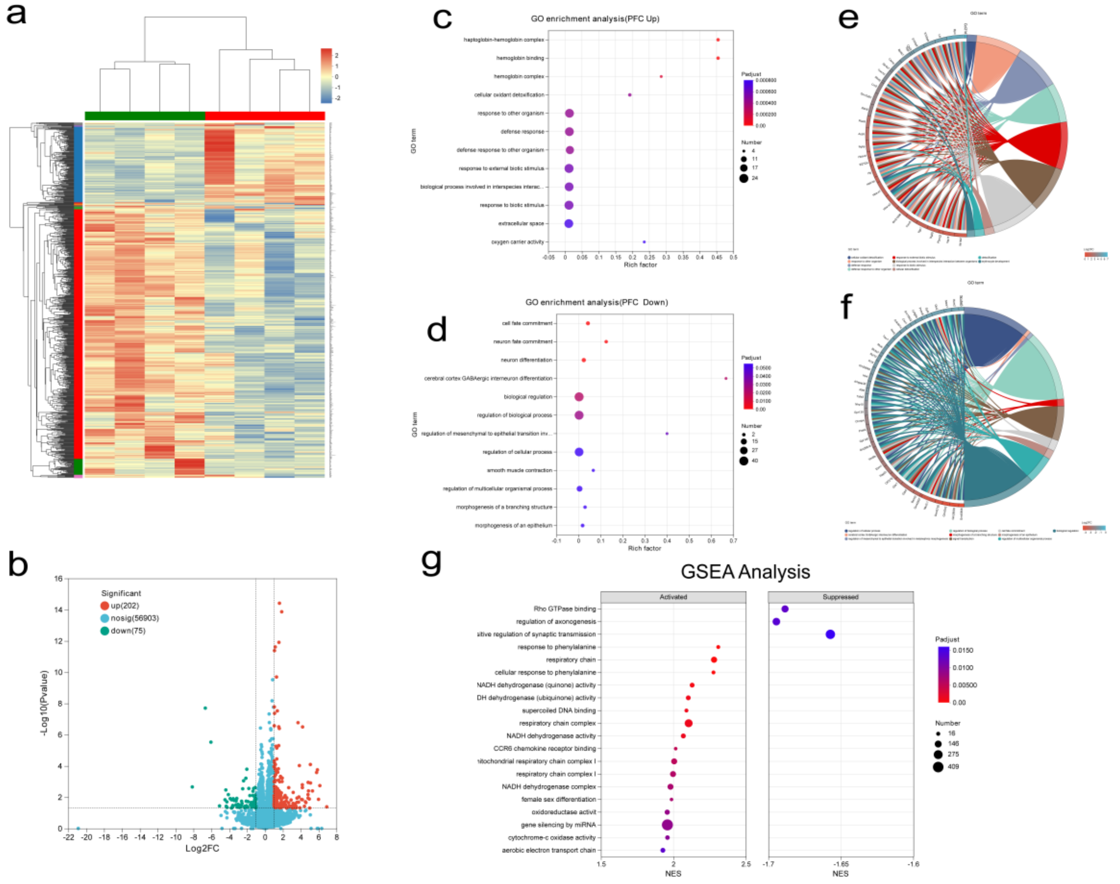

Supplementary Fig 3. Transcriptional Characteristics of the PFC. a Clustering heatmap of PFC transcriptome data, showing significant expression differences between the Flea- and Flea+ groups. b Volcano plot of differentially expressed genes in the PFC transcriptome. Red indicates upregulated genes in the Flea+ group, while green indicates downregulated genes. c-d Gene ontology terms enriched in genes that are upregulated (c) or downregulated (d) in the PFC in Flea+ relative to Flea- mice. e-f Chord diagram of the top 10 significantly different genes in upregulated (e) or downregulated (f) enriched functions. g Significantly enriched functions from GSEA analysis of the PFC transcriptome: activated functions on the left, suppressed functions on the right, relative to Flea- group.

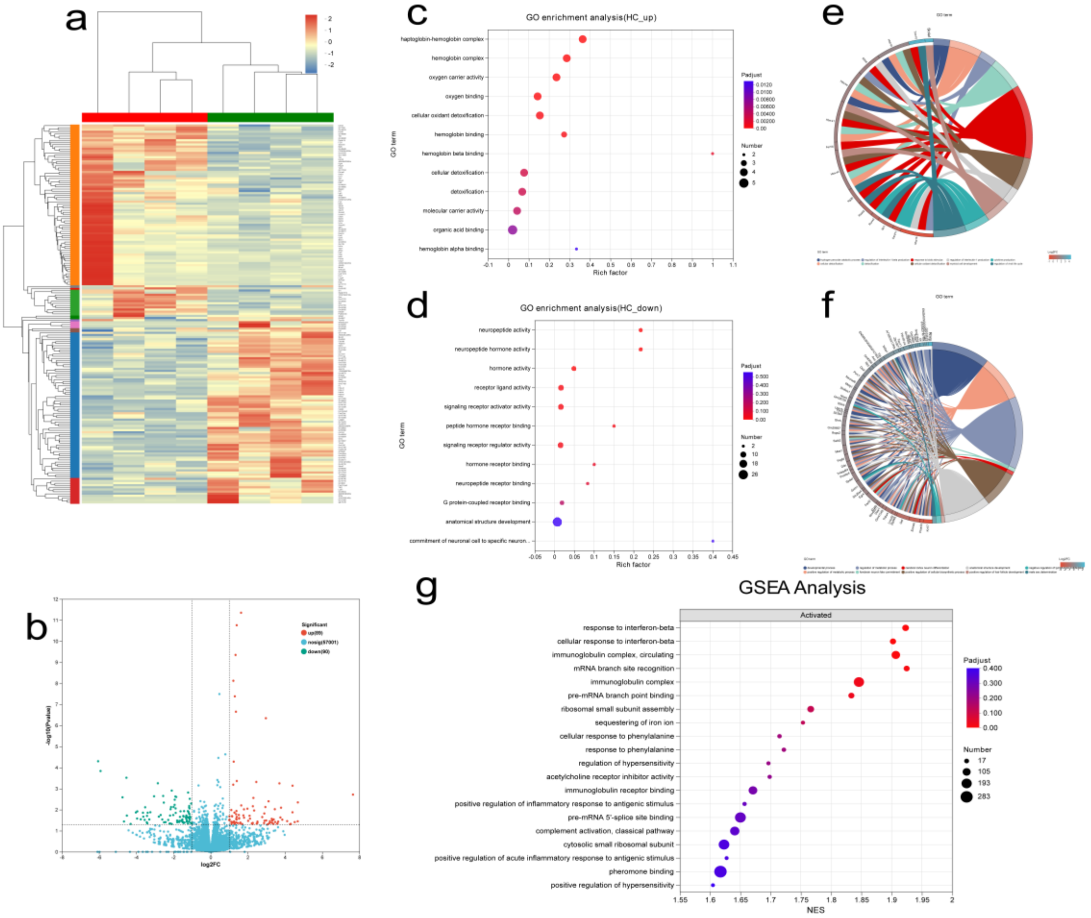

Supplementary Fig 4. Transcriptional Characteristics of the HC. a Clustering heatmap of HC transcriptome data, showing significant expression differences between the Flea- and Flea+ groups. b Volcano plot of differentially expressed genes in the HC transcriptome. Red indicates upregulated genes in the Flea+ group, while green indicates downregulated genes. c-d Gene ontology terms enriched in genes that are upregulated (c) or downregulated (d) in the HC in Flea+ relative to Flea- mice. e-f Chord diagram of the top 10 significantly different genes in upregulated (e) or downregulated (f) enriched functions. g Significantly enriched functions from GSEA analysis of the HC transcriptome: activated functions on the left, suppressed functions on the right, relative to Flea- group.

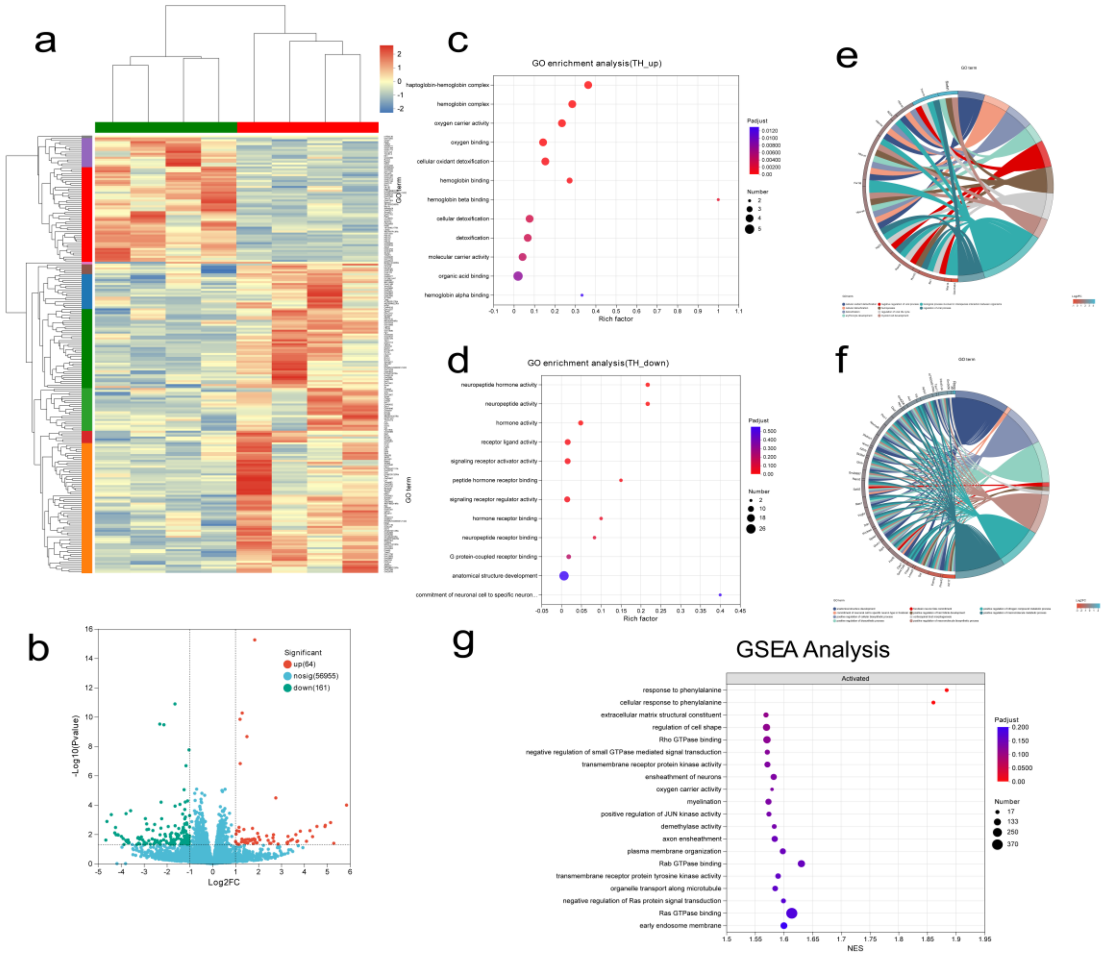

Supplementary Fig 5. Transcriptional Characteristics of the TH. a Clustering heatmap of TH transcriptome data, showing significant expression differences between the Flea- and Flea+ groups. b Volcano plot of differentially expressed genes in the TH transcriptome. Red indicates upregulated genes in the Flea+ group, while green indicates downregulated genes. c-d Gene ontology terms enriched in genes that are upregulated (c) or downregulated (d) in the TH in Flea+ relative to Flea- mice. e-f Chord diagram of the top 10 significantly different genes in upregulated (e) or downregulated (f) enriched functions. g Significantly enriched functions from GSEA analysis of the HC transcriptome: activated functions on the left, suppressed functions on the right, relative to Flea- group.

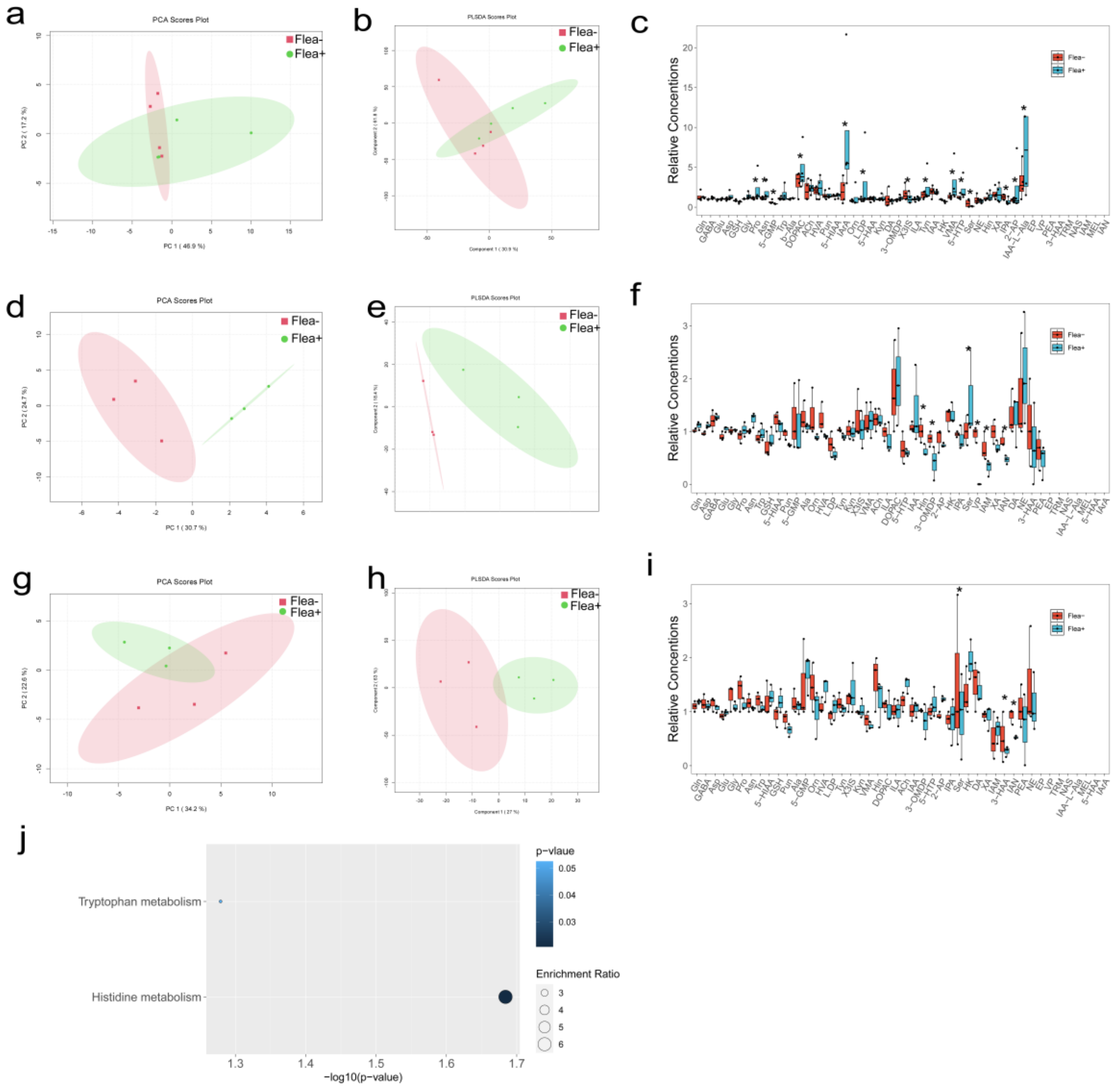

Supplementary Fig 6. Neurotransmitter profiling of the PFC reveals altered pathways related to emotional regulation. a PCA of PFC neurotransmitter metabolomics reveals group-specific clustering, with each region color-coded according to the legend. b PLSDA plot of PFC neurotransmitter metabolomics. c Neurotransmitter metabolomics levels in the PFC presented in boxplot, where red is Flea- (n=4), and blue is Flea+ (n=4). Two-sided t test was used. Thick bars indicate the interquartile range (IQR) around the median, and whiskers represent 1.5 times the interquartile range (maxima: Q3 + 1.5*IQR, minima: Q1 − 1.5*IQR). Each black dot represents pooled sample of 3 decapitated individuals. Asterisks indicate the significance. d PCA of HC neurotransmitter metabolomics. e PLSDA plot of HC neurotransmitter metabolomics. f Neurotransmitter metabolomics levels in the HC presented in boxplot, where red is Flea- (n=3), and blue is Flea+ (n=3). Two-sided t test was used. Thick bars indicate the interquartile range (IQR) around the median, and whiskers represent 1.5 times the interquartile range (maxima: Q3 + 1.5*IQR, minima: Q1 − 1.5*IQR). Each black dot represents pooled sample of 3 decapitated individuals. Asterisks indicate the significance. g PCA of TH neurotransmitter metabolomics. h PLSDA plot of TH

neurotransmitter metabolomics. i Neurotransmitter metabolomics levels in the TH presented in boxplot, where red is Flea- (n=3), and blue is Flea+ (n=3). Two-sided t test was used. Thick bars indicate the interquartile range (IQR) around the median, and whiskers represent 1.5 times the interquartile range (maxima: Q3 + 1.5*IQR, minima: Q1 − 1.5*IQR). Each black dot represents pooled sample of 3 decapitated individuals. Asterisks indicate the significance. j KEGG pathway enrichment analysis of differential neurotransmitters in the HC. Source data are provided as a Source Data file. $**p < 0.01$, $*p < 0.05$

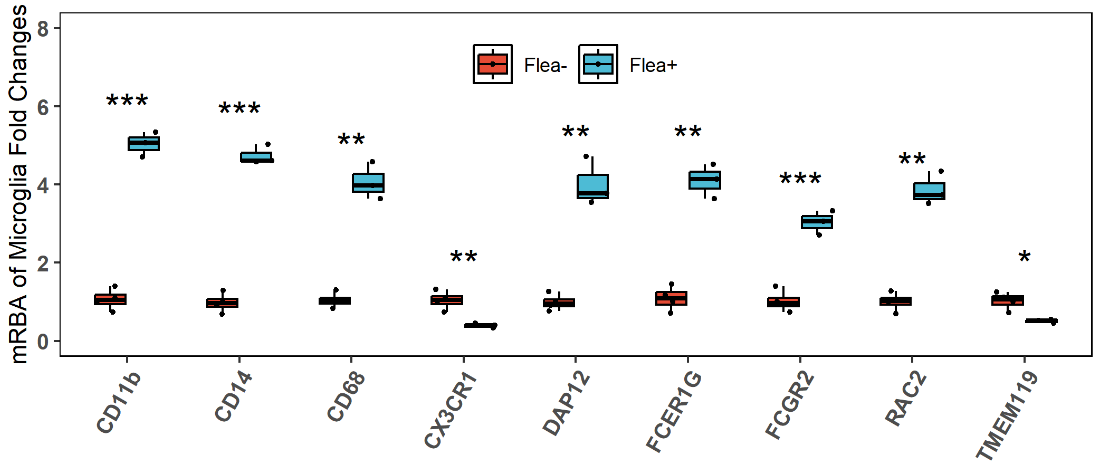

Supplementary Fig 7. Validation by qPCR of select genes altered between Flea- (n=3) and Flea+ (n=3) group. Two-sided t test was used. Each black dot represents pooled sample of 3 decapitated individuals. Asterisks indicate the significance. Source data are provided as a Source Data file. $***p < 0.001$, $**p < 0.01$, $*p < 0.05$

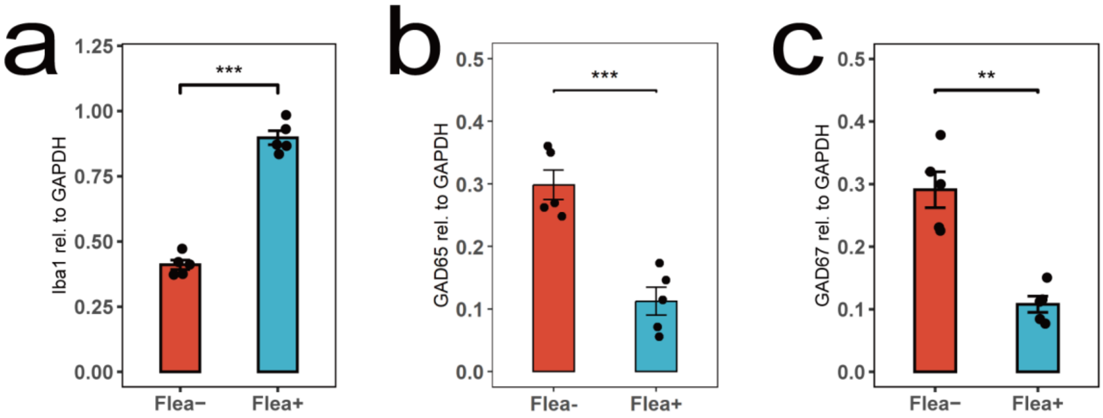

Supplementary Fig 8. Statistical comparison of groups based on Western blot results. a IBA1. b GAD65. c GAD67. Comparison of IBA1, Gad65, and GAD67 between the Flea- (red, n=5) and Flea+ (blue, n=5) groups of mice. Two-sided t test was used. Each black dot represents an individual. Asterisks indicates brain regions with significant differences. Data are presented as mean ± SEM. Source data are provided as a Source

Data file. ***$p < 0.001$, **$p < 0.01$

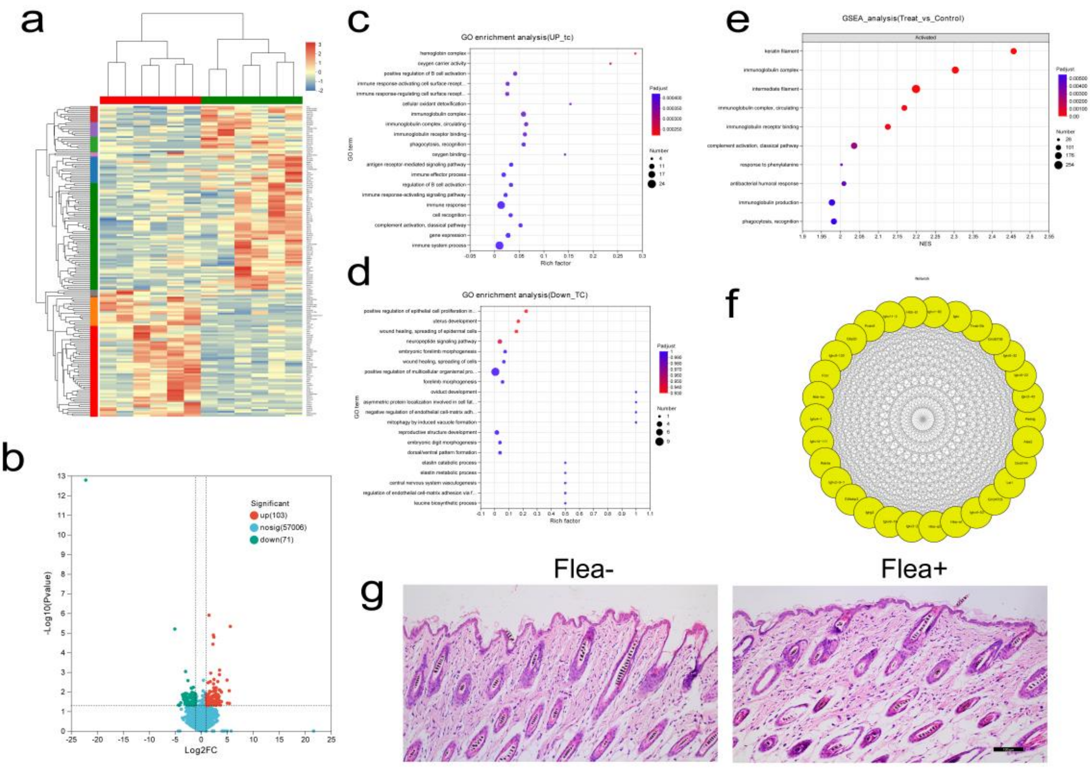

Supplementary Fig 9. Transcriptional Characteristics of the skin. a Clustering heatmap of skin transcriptome data, showing significant expression differences between the Flea- and Flea+ groups. b Volcano plot of differentially expressed genes in the skin transcriptome. Red indicates upregulated genes in the Flea+ group, while green indicates downregulated genes. c-d Gene ontology terms enriched in genes that are upregulated (c) or downregulated (d) in the skin in Flea+ relative to Flea- mice. e Significantly enriched functions from GSEA analysis of the HC transcriptome: activated functions on the left, suppressed functions on the right, relative to Flea- group. f The protein-protein interaction (PPI) network of the skin transcriptome shows that genes associated with flea bites are primarily related to immunoglobulins (Ig) and hemoglobin (Hb). g HE staining of mice skin revealed no significant pathological damage in the Flea+ group.

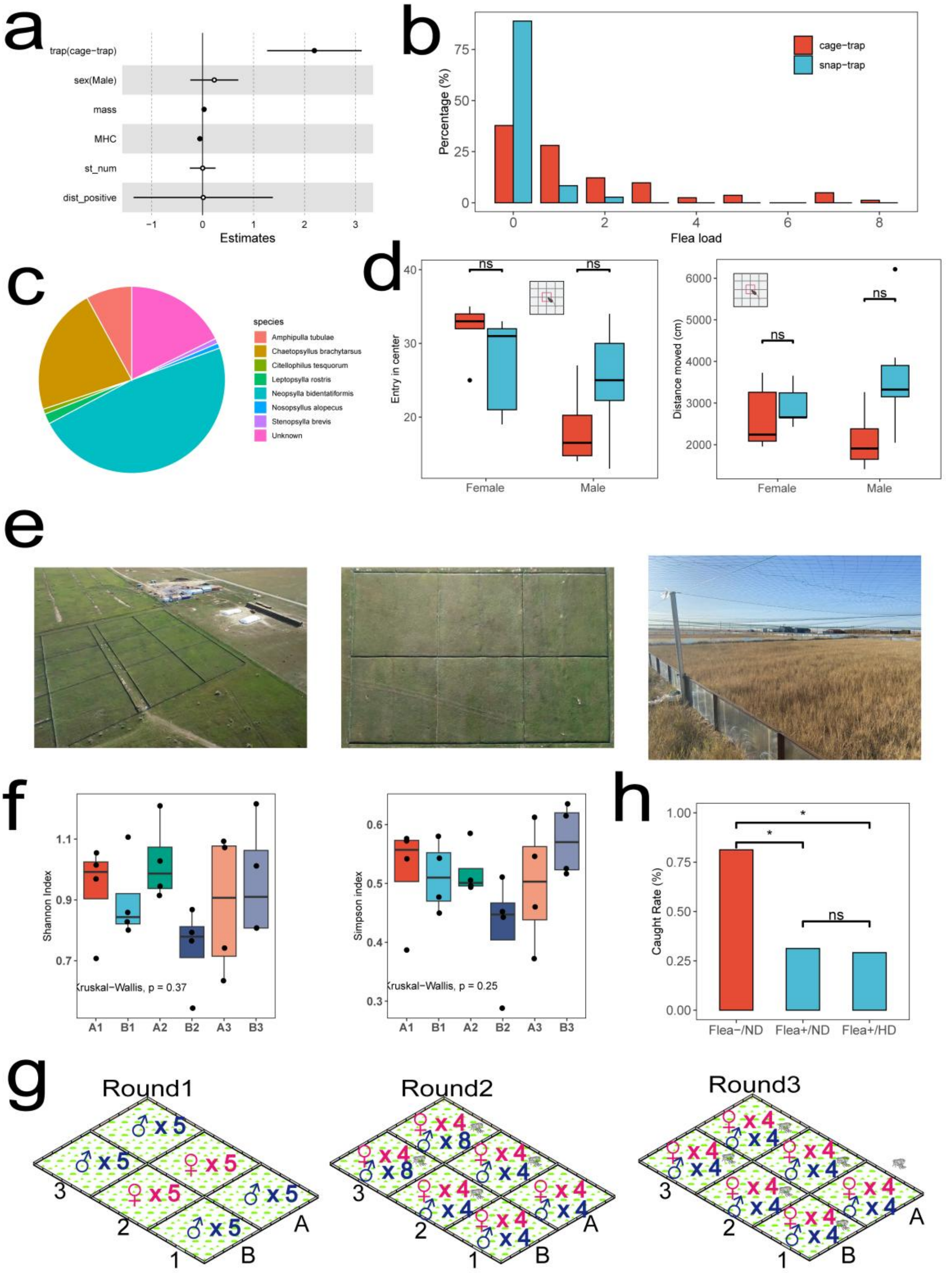

Supplementary Fig 10. Striped hamsters subjected to flea bites exhibited a decrease in exploratory behavior under both laboratory conditions and in the outdoor enclosure experiment. a Forest plot illustrating factors influencing flea load in wild striped hamsters, tested using a Generalized Linear Model (GLM). The x-axis represents the coefficients of various predictor variables. Solid circles indicate statistically significant results, while hollow circles represent non-significant results. Variables such as capture method, mass, and the number of MHC alleles are shown to be associated with flea load

in wild striped hamsters. b Cage-captured striped hamsters had higher flea load and infestation rates than trap-captured ones. c *Neopsylla bidentatiformis* was the dominant flea species on striped hamsters in the W4 site. d No significant differences in center entries (left) or distance traveled (right) were observed between the groups for both male and female striped hamsters. One-sided t test was used. ns, not significant. e Photograph of the enclosure used for controlled experiments on the Xilingol Grassland in Inner Mongolia. f Vegetation diversity assessed through sampling methods showed no differences in both Shannon (left) and Simpson indices (right). g Schematic diagram of the three-round enclosure experimental design. h Capture rates for striped hamsters in the Round2 enclosure experiment. Flea+ hamsters had a lower capture rate. Source data are provided as a Source Data file.

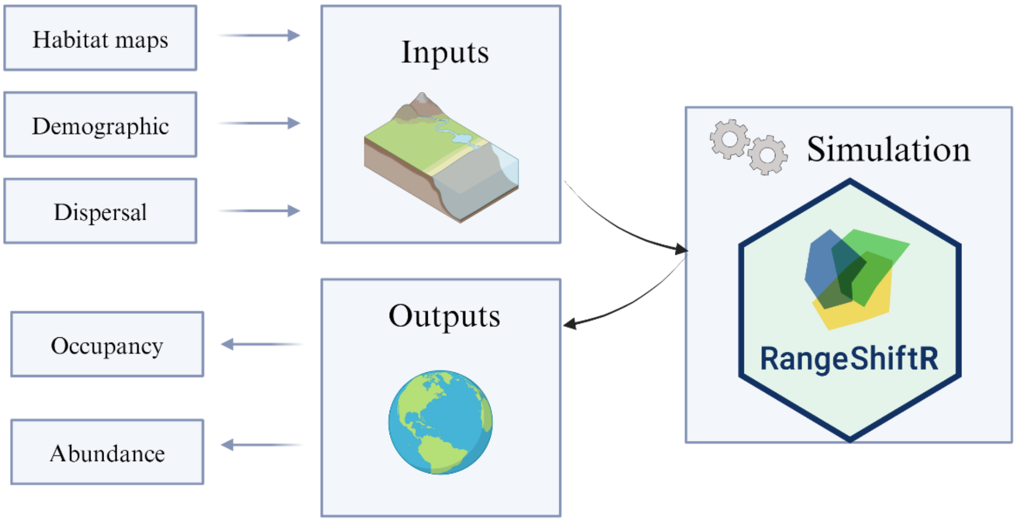

Supplementary Fig 11. The workflow diagram for the individual-based mechanism model RangeshiftR includes the following steps. (1) Using environmental variables and species distribution data, species distribution models are created to establish the current and future suitable habitats for black-striped hamsters. (2) Demographic data for black-striped hamsters are gathered from three years of field sampling and literature records. (3) Data on the dispersal characteristics of black-striped hamsters are obtained through semi-natural enclosure experiments and literature review. (4) RangeshiftR simulations generate outputs detailing the distribution and density of black-striped hamsters under various parasite infection scenarios

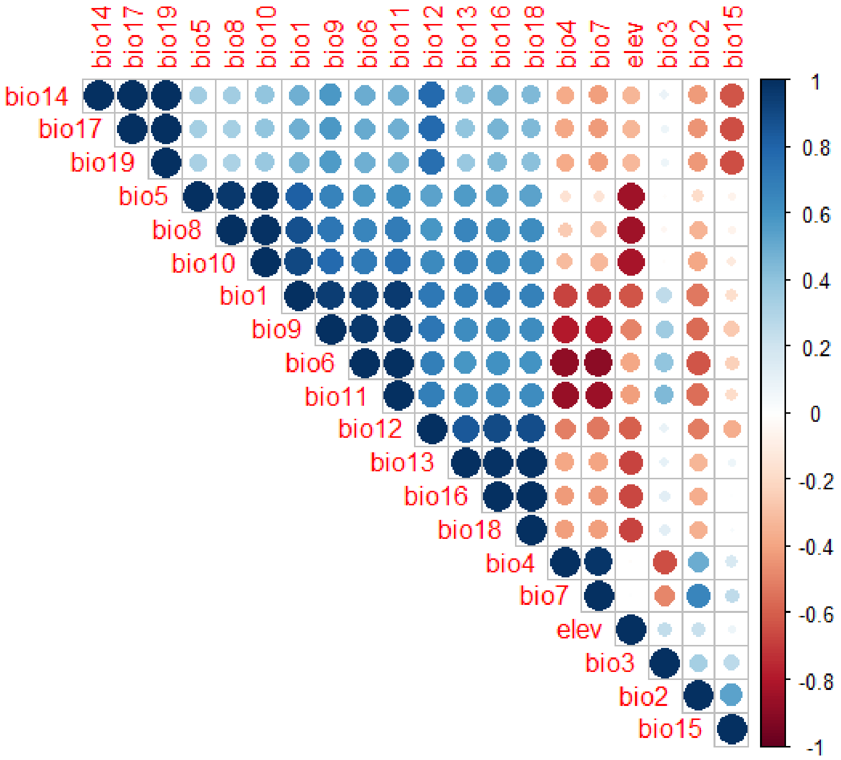

Supplementary Fig 12. Spearman correlation between environmental variables predicting suitable habitat for striped hamsters. Two-sided t test was used. Source data are provided as a Source Data file.

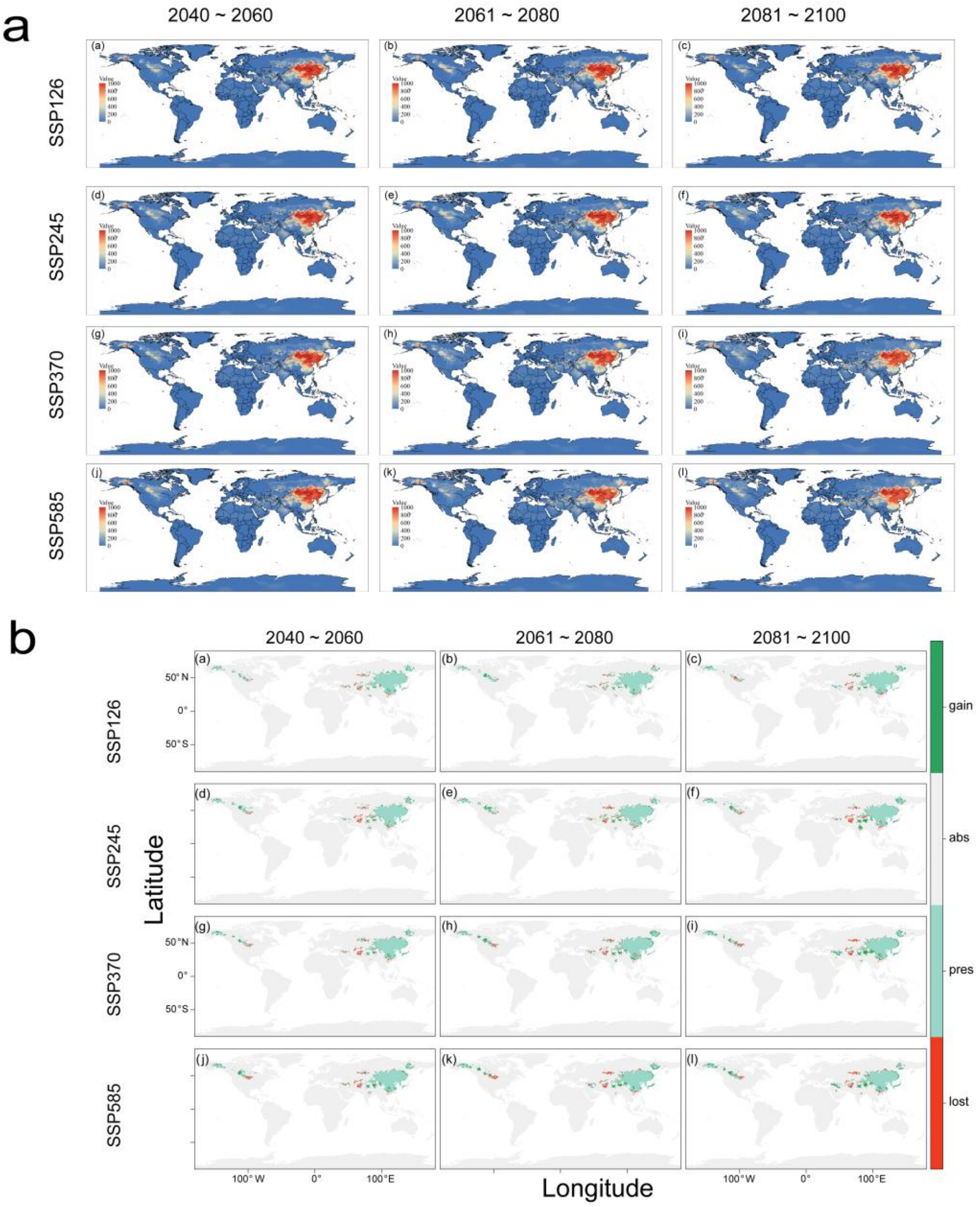

Supplementary Fig 13. a Global habitat suitability distribution for striped hamsters under future climate scenarios. b Global changes in habitat suitability for striped hamsters under future climate scenarios.

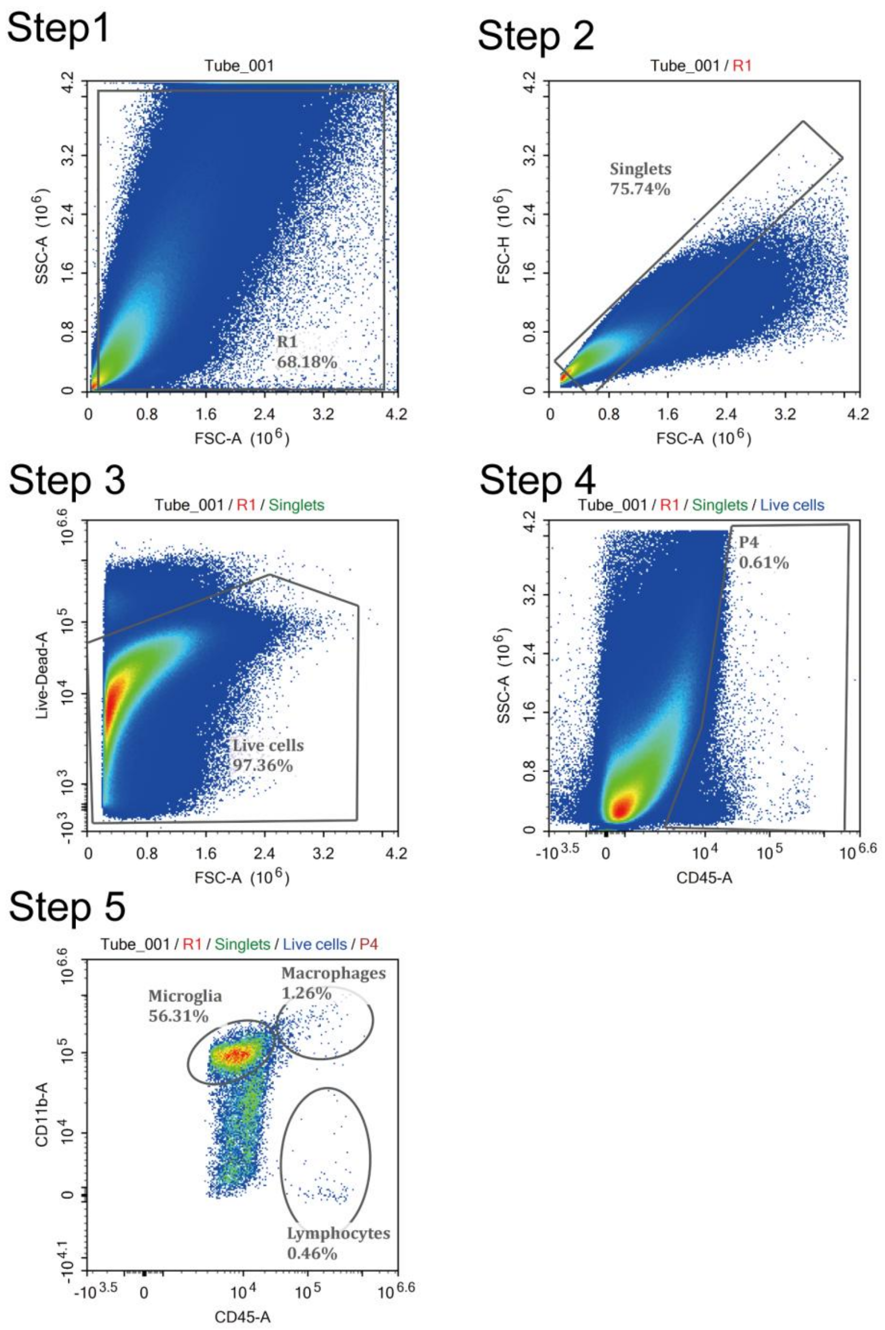

Supplementary Fig 14. Flow cytometry gating strategies used in the paper were applied to samples from the Flea^{-} group. Specifically, the final gating panel corresponds to Figure 3h in the main manuscript.

Supplementary Table 1. 562 cleaned occurrence sites of striped hamster

No.	Species	Longitude	Latitude	Country
1	*Cricetulus barabensis*	105.88	46.90	Mongolia
2	*Cricetulus barabensis*	103.66	47.40	Mongolia
3	*Cricetulus barabensis*	103.65	47.40	Mongolia
4	*Cricetulus barabensis*	103.65	47.40	Mongolia
5	*Cricetulus barabensis*	103.65	47.40	Mongolia
6	*Cricetulus barabensis*	103.65	47.40	Mongolia
7	*Cricetulus barabensis*	103.66	47.40	Mongolia
8	*Cricetulus barabensis*	103.66	47.40	Mongolia
9	*Cricetulus barabensis*	103.66	47.40	Mongolia
10	*Cricetulus barabensis*	103.66	47.40	Mongolia
11	*Cricetulus barabensis*	103.66	47.40	Mongolia
12	*Cricetulus barabensis*	103.66	47.40	Mongolia
13	*Cricetulus barabensis*	103.66	47.40	Mongolia
14	*Cricetulus barabensis*	103.66	47.40	Mongolia
15	*Cricetulus barabensis*	103.66	47.40	Mongolia
16	*Cricetulus barabensis*	103.66	47.40	Mongolia
17	*Cricetulus barabensis*	103.66	47.40	Mongolia
18	*Cricetulus barabensis*	103.66	47.40	Mongolia
19	*Cricetulus barabensis*	103.65	47.40	Mongolia
20	*Cricetulus barabensis*	103.65	47.40	Mongolia
21	*Cricetulus barabensis*	103.65	47.40	Mongolia
22	*Cricetulus barabensis*	103.65	47.40	Mongolia
23	*Cricetulus barabensis*	103.66	47.40	Mongolia
24	*Cricetulus barabensis*	103.65	47.40	Mongolia
25	*Cricetulus barabensis*	106.25	47.58	Mongolia
26	*Cricetulus barabensis*	106.25	47.57	Mongolia
27	*Cricetulus barabensis*	106.25	47.58	Mongolia
28	*Cricetulus barabensis*	106.25	47.57	Mongolia
29	*Cricetulus barabensis*	106.25	47.58	Mongolia
30	*Cricetulus barabensis*	106.25	47.58	Mongolia
31	*Cricetulus barabensis*	106.25	47.57	Mongolia
32	*Cricetulus barabensis*	106.25	47.58	Mongolia
33	*Cricetulus barabensis*	106.25	47.58	Mongolia
34	*Cricetulus barabensis*	106.25	47.58	Mongolia
35	*Cricetulus barabensis*	106.25	47.58	Mongolia
36	*Cricetulus barabensis*	106.25	47.57	Mongolia
37	*Cricetulus barabensis*	106.25	47.58	Mongolia
38	*Cricetulus barabensis*	106.25	47.58	Mongolia
39	*Cricetulus barabensis*	106.26	47.58	Mongolia
40	*Cricetulus barabensis*	106.25	47.57	Mongolia
41	*Cricetulus barabensis*	106.25	47.58	Mongolia

42	*Cricetulus barabensis*	106.25	47.57	Mongolia
43	*Cricetulus barabensis*	106.25	47.58	Mongolia
44	*Cricetulus barabensis*	106.25	47.57	Mongolia
45	*Cricetulus barabensis*	106.25	47.58	Mongolia
46	*Cricetulus barabensis*	106.25	47.57	Mongolia
47	*Cricetulus barabensis*	106.25	47.57	Mongolia
48	*Cricetulus barabensis*	106.25	47.58	Mongolia
49	*Cricetulus barabensis*	102.05	46.77	Mongolia
50	*Cricetulus barabensis*	102.02	46.80	Mongolia
51	*Cricetulus barabensis*	102.05	46.77	Mongolia
52	*Cricetulus barabensis*	102.02	46.80	Mongolia
53	*Cricetulus barabensis*	102.06	46.76	Mongolia
54	*Cricetulus barabensis*	108.99	47.43	Mongolia
55	*Cricetulus barabensis*	110.23	48.40	Mongolia
56	*Cricetulus barabensis*	108.95	47.45	Mongolia
57	*Cricetulus barabensis*	108.95	47.45	Mongolia
58	*Cricetulus barabensis*	108.99	47.43	Mongolia
59	*Cricetulus barabensis*	108.95	47.45	Mongolia
60	*Cricetulus barabensis*	108.98	47.43	Mongolia
61	*Cricetulus barabensis*	108.95	47.45	Mongolia
62	*Cricetulus barabensis*	110.22	48.40	Mongolia
63	*Cricetulus barabensis*	110.24	48.40	Mongolia
64	*Cricetulus barabensis*	110.23	48.40	Mongolia
65	*Cricetulus barabensis*	110.23	48.40	Mongolia
66	*Cricetulus barabensis*	110.23	48.40	Mongolia
67	*Cricetulus barabensis*	108.99	47.43	Mongolia
68	*Cricetulus barabensis*	108.99	47.43	Mongolia
69	*Cricetulus barabensis*	108.98	47.43	Mongolia
70	*Cricetulus barabensis*	110.24	48.40	Mongolia
71	*Cricetulus barabensis*	110.23	48.40	Mongolia
72	*Cricetulus barabensis*	110.23	48.40	Mongolia
73	*Cricetulus barabensis*	110.23	48.40	Mongolia
74	*Cricetulus barabensis*	110.24	48.40	Mongolia
75	*Cricetulus barabensis*	110.23	48.40	Mongolia
76	*Cricetulus barabensis*	110.24	48.40	Mongolia
77	*Cricetulus barabensis*	108.98	47.43	Mongolia
78	*Cricetulus barabensis*	108.98	47.43	Mongolia
79	*Cricetulus barabensis*	108.98	47.43	Mongolia
80	*Cricetulus barabensis*	108.98	47.43	Mongolia
81	*Cricetulus barabensis*	107.62	47.77	Mongolia
82	*Cricetulus barabensis*	107.62	47.77	Mongolia
83	*Cricetulus barabensis*	107.62	47.77	Mongolia
84	*Cricetulus barabensis*	107.62	47.77	Mongolia
85	*Cricetulus barabensis*	107.62	47.78	Mongolia

86	*Cricetulus barabensis*	109.85	48.12	Mongolia
87	*Cricetulus barabensis*	109.85	48.13	Mongolia
88	*Cricetulus barabensis*	109.85	48.13	Mongolia
89	*Cricetulus barabensis*	109.86	48.12	Mongolia
90	*Cricetulus barabensis*	109.85	48.13	Mongolia
91	*Cricetulus barabensis*	109.85	48.13	Mongolia
92	*Cricetulus barabensis*	109.85	48.13	Mongolia
93	*Cricetulus barabensis*	110.31	48.70	Mongolia
94	*Cricetulus barabensis*	110.32	48.70	Mongolia
95	*Cricetulus barabensis*	107.62	47.77	Mongolia
96	*Cricetulus barabensis*	109.85	48.13	Mongolia
97	*Cricetulus barabensis*	110.32	48.70	Mongolia
98	*Cricetulus barabensis*	103.57	49.78	Mongolia
99	*Cricetulus barabensis*	103.27	49.78	Mongolia
100	*Cricetulus barabensis*	103.27	49.78	Mongolia
101	*Cricetulus barabensis*	103.27	49.78	Mongolia
102	*Cricetulus barabensis*	103.27	49.78	Mongolia
103	*Cricetulus barabensis*	103.27	49.78	Mongolia
104	*Cricetulus barabensis*	103.27	49.78	Mongolia
105	*Cricetulus barabensis*	103.27	49.78	Mongolia
106	*Cricetulus barabensis*	103.27	49.78	Mongolia
107	*Cricetulus barabensis*	105.21	49.16	Mongolia
108	*Cricetulus barabensis*	105.22	49.16	Mongolia
109	*Cricetulus barabensis*	105.21	49.16	Mongolia
110	*Cricetulus barabensis*	105.21	49.16	Mongolia
111	*Cricetulus barabensis*	105.22	49.16	Mongolia
112	*Cricetulus barabensis*	105.22	49.16	Mongolia
113	*Cricetulus barabensis*	110.31	48.70	Mongolia
114	*Cricetulus barabensis*	110.66	48.77	Mongolia
115	*Cricetulus barabensis*	110.66	48.77	Mongolia
116	*Cricetulus barabensis*	110.66	48.77	Mongolia
117	*Cricetulus barabensis*	110.66	48.77	Mongolia
118	*Cricetulus barabensis*	110.66	48.77	Mongolia
119	*Cricetulus barabensis*	110.66	48.77	Mongolia
120	*Cricetulus barabensis*	110.67	48.77	Mongolia
121	*Cricetulus barabensis*	110.66	48.78	Mongolia
122	*Cricetulus barabensis*	110.66	48.78	Mongolia
123	*Cricetulus barabensis*	119.38	47.01	Mongolia
124	*Cricetulus barabensis*	119.38	47.01	Mongolia
125	*Cricetulus barabensis*	119.37	47.00	Mongolia
126	*Cricetulus barabensis*	119.38	47.01	Mongolia
127	*Cricetulus barabensis*	119.37	47.00	Mongolia
128	*Cricetulus barabensis*	119.37	47.00	Mongolia
129	*Cricetulus barabensis*	119.37	47.00	Mongolia

130	*Cricetulus barabensis*	119.37	47.00	Mongolia
131	*Cricetulus barabensis*	119.37	47.00	Mongolia
132	*Cricetulus barabensis*	111.88	49.12	Mongolia
133	*Cricetulus barabensis*	113.54	48.20	Mongolia
134	*Cricetulus barabensis*	113.54	48.20	Mongolia
135	*Cricetulus barabensis*	113.54	48.20	Mongolia
136	*Cricetulus barabensis*	113.54	48.20	Mongolia
137	*Cricetulus barabensis*	113.54	48.20	Mongolia
138	*Cricetulus barabensis*	113.54	48.20	Mongolia
139	*Cricetulus barabensis*	113.54	48.20	Mongolia
140	*Cricetulus barabensis*	113.54	48.20	Mongolia
141	*Cricetulus barabensis*	113.54	48.20	Mongolia
142	*Cricetulus barabensis*	113.54	48.20	Mongolia
143	*Cricetulus barabensis*	119.38	47.01	Mongolia
144	*Cricetulus barabensis*	119.38	47.01	Mongolia
145	*Cricetulus barabensis*	119.37	47.00	Mongolia
146	*Cricetulus barabensis*	119.38	47.00	Mongolia
147	*Cricetulus barabensis*	119.38	47.00	Mongolia
148	*Cricetulus barabensis*	119.38	47.01	Mongolia
149	*Cricetulus barabensis*	119.38	47.00	Mongolia
150	*Cricetulus barabensis*	119.37	46.99	Mongolia
151	*Cricetulus barabensis*	119.37	46.99	Mongolia
152	*Cricetulus barabensis*	119.36	46.98	Mongolia
153	*Cricetulus barabensis*	119.37	46.99	Mongolia
154	*Cricetulus barabensis*	119.37	46.99	Mongolia
155	*Cricetulus barabensis*	119.38	47.01	Mongolia
156	*Cricetulus barabensis*	119.16	47.12	Mongolia
157	*Cricetulus barabensis*	119.16	47.10	Mongolia
158	*Cricetulus barabensis*	119.17	47.11	Mongolia
159	*Cricetulus barabensis*	119.17	47.11	Mongolia
160	*Cricetulus barabensis*	119.16	47.10	Mongolia
161	*Cricetulus barabensis*	119.16	47.10	Mongolia
162	*Cricetulus barabensis*	119.16	47.12	Mongolia
163	*Cricetulus barabensis*	119.16	47.10	Mongolia
164	*Cricetulus barabensis*	119.16	47.12	Mongolia
165	*Cricetulus barabensis*	119.16	47.10	Mongolia
166	*Cricetulus barabensis*	119.16	47.12	Mongolia
167	*Cricetulus barabensis*	119.16	47.10	Mongolia
168	*Cricetulus barabensis*	119.15	47.10	Mongolia
169	*Cricetulus barabensis*	119.16	47.10	Mongolia
170	*Cricetulus barabensis*	119.16	47.10	Mongolia
171	*Cricetulus barabensis*	119.16	47.10	Mongolia
172	*Cricetulus barabensis*	119.15	47.11	Mongolia
173	*Cricetulus barabensis*	119.16	47.11	Mongolia

174	*Cricetulus barabensis*	119.16	47.12	Mongolia
175	*Cricetulus barabensis*	119.16	47.12	Mongolia
176	*Cricetulus barabensis*	119.16	47.12	Mongolia
177	*Cricetulus barabensis*	119.16	47.10	Mongolia
178	*Cricetulus barabensis*	119.16	47.10	Mongolia
179	*Cricetulus barabensis*	119.16	47.10	Mongolia
180	*Cricetulus barabensis*	119.16	47.12	Mongolia
181	*Cricetulus barabensis*	119.16	47.10	Mongolia
182	*Cricetulus barabensis*	119.16	47.10	Mongolia
183	*Cricetulus barabensis*	119.16	47.10	Mongolia
184	*Cricetulus barabensis*	119.16	47.10	Mongolia
185	*Cricetulus barabensis*	119.16	47.10	Mongolia
186	*Cricetulus barabensis*	119.16	47.10	Mongolia
187	*Cricetulus barabensis*	119.16	47.10	Mongolia
188	*Cricetulus barabensis*	119.16	47.10	Mongolia
189	*Cricetulus barabensis*	119.16	47.10	Mongolia
190	*Cricetulus barabensis*	119.17	47.11	Mongolia
191	*Cricetulus barabensis*	119.17	47.11	Mongolia
192	*Cricetulus barabensis*	119.16	47.10	Mongolia
193	*Cricetulus barabensis*	119.16	47.10	Mongolia
194	*Cricetulus barabensis*	119.16	47.10	Mongolia
195	*Cricetulus barabensis*	119.16	47.10	Mongolia
196	*Cricetulus barabensis*	119.16	47.10	Mongolia
197	*Cricetulus barabensis*	119.16	47.10	Mongolia
198	*Cricetulus barabensis*	119.15	47.10	Mongolia
199	*Cricetulus barabensis*	119.16	47.10	Mongolia
200	*Cricetulus barabensis*	119.17	47.11	Mongolia
201	*Cricetulus barabensis*	119.16	47.10	Mongolia
202	*Cricetulus barabensis*	119.16	47.10	Mongolia
203	*Cricetulus barabensis*	119.16	47.10	Mongolia
204	*Cricetulus barabensis*	118.95	47.27	Mongolia
205	*Cricetulus barabensis*	118.95	47.27	Mongolia
206	*Cricetulus barabensis*	118.95	47.27	Mongolia
207	*Cricetulus barabensis*	118.95	47.27	Mongolia
208	*Cricetulus barabensis*	119.16	47.10	Mongolia
209	*Cricetulus barabensis*	118.95	47.27	Mongolia
210	*Cricetulus barabensis*	118.94	47.27	Mongolia
211	*Cricetulus barabensis*	118.94	47.27	Mongolia
212	*Cricetulus barabensis*	118.94	47.27	Mongolia
213	*Cricetulus barabensis*	118.95	47.27	Mongolia
214	*Cricetulus barabensis*	118.95	47.27	Mongolia
215	*Cricetulus barabensis*	118.94	47.27	Mongolia
216	*Cricetulus barabensis*	118.94	47.27	Mongolia
217	*Cricetulus barabensis*	118.94	47.27	Mongolia

218	*Cricetulus barabensis*	118.95	47.27	Mongolia
219	*Cricetulus barabensis*	118.94	47.27	Mongolia
220	*Cricetulus barabensis*	118.94	47.27	Mongolia
221	*Cricetulus barabensis*	118.95	47.27	Mongolia
222	*Cricetulus barabensis*	118.95	47.27	Mongolia
223	*Cricetulus barabensis*	118.95	47.27	Mongolia
224	*Cricetulus barabensis*	118.95	47.27	Mongolia
225	*Cricetulus barabensis*	118.94	47.27	Mongolia
226	*Cricetulus barabensis*	118.94	47.27	Mongolia
227	*Cricetulus barabensis*	118.95	47.27	Mongolia
228	*Cricetulus barabensis*	118.94	47.27	Mongolia
229	*Cricetulus barabensis*	118.94	47.27	Mongolia
230	*Cricetulus barabensis*	118.95	47.27	Mongolia
231	*Cricetulus barabensis*	118.95	47.27	Mongolia
232	*Cricetulus barabensis*	118.95	47.27	Mongolia
233	*Cricetulus barabensis*	118.94	47.27	Mongolia
234	*Cricetulus barabensis*	118.94	47.27	Mongolia
235	*Cricetulus barabensis*	119.37	47.00	Mongolia
236	*Cricetulus barabensis*	119.37	47.00	Mongolia
237	*Cricetulus barabensis*	119.38	47.01	Mongolia
238	*Cricetulus barabensis*	119.37	47.00	Mongolia
239	*Cricetulus barabensis*	119.38	47.01	Mongolia
240	*Cricetulus barabensis*	119.38	47.01	Mongolia
241	*Cricetulus barabensis*	119.37	47.00	Mongolia
242	*Cricetulus barabensis*	119.38	47.01	Mongolia
243	*Cricetulus barabensis*	119.37	47.00	Mongolia
244	*Cricetulus barabensis*	119.37	47.00	Mongolia
245	*Cricetulus barabensis*	119.37	47.00	Mongolia
246	*Cricetulus barabensis*	119.37	47.00	Mongolia
247	*Cricetulus barabensis*	119.37	47.00	Mongolia
248	*Cricetulus barabensis*	119.37	47.00	Mongolia
249	*Cricetulus barabensis*	119.37	47.00	Mongolia
250	*Cricetulus barabensis*	119.37	47.00	Mongolia
251	*Cricetulus barabensis*	119.37	47.01	Mongolia
252	*Cricetulus barabensis*	119.37	47.00	Mongolia
253	*Cricetulus barabensis*	119.37	47.00	Mongolia
254	*Cricetulus barabensis*	119.37	47.00	Mongolia
255	*Cricetulus barabensis*	119.37	47.00	Mongolia
256	*Cricetulus barabensis*	119.37	47.00	Mongolia
257	*Cricetulus barabensis*	119.37	47.00	Mongolia
258	*Cricetulus barabensis*	119.37	47.01	Mongolia
259	*Cricetulus barabensis*	119.37	47.00	Mongolia
260	*Cricetulus barabensis*	119.37	46.99	Mongolia
261	*Cricetulus barabensis*	119.38	47.01	Mongolia

262	*Cricetulus barabensis*	119.37	47.00	Mongolia
263	*Cricetulus barabensis*	119.37	47.00	Mongolia
264	*Cricetulus barabensis*	119.37	47.00	Mongolia
265	*Cricetulus barabensis*	119.37	47.00	Mongolia
266	*Cricetulus barabensis*	119.37	47.00	Mongolia
267	*Cricetulus barabensis*	119.38	47.01	Mongolia
268	*Cricetulus barabensis*	119.38	47.01	Mongolia
269	*Cricetulus barabensis*	119.38	47.01	Mongolia
270	*Cricetulus barabensis*	119.38	47.01	Mongolia
271	*Cricetulus barabensis*	119.37	47.00	Mongolia
272	*Cricetulus barabensis*	119.37	47.00	Mongolia
273	*Cricetulus barabensis*	119.37	47.00	Mongolia
274	*Cricetulus barabensis*	119.37	46.99	Mongolia
275	*Cricetulus barabensis*	100.09	48.13	Mongolia
276	*Cricetulus barabensis*	100.09	48.13	Mongolia
277	*Cricetulus barabensis*	100.09	48.13	Mongolia
278	*Cricetulus barabensis*	100.09	48.13	Mongolia
279	*Cricetulus barabensis*	100.09	48.13	Mongolia
280	*Cricetulus barabensis*	100.53	50.54	Mongolia
281	*Cricetulus barabensis*	100.53	50.54	Mongolia
282	*Cricetulus barabensis*	91.65	50.04	Mongolia
283	*Cricetulus barabensis*	94.65	49.45	Mongolia
284	*Cricetulus barabensis*	94.65	49.46	Mongolia
285	*Cricetulus barabensis*	94.65	49.45	Mongolia
286	*Cricetulus barabensis*	94.65	49.45	Mongolia
287	*Cricetulus barabensis*	94.65	49.45	Mongolia
288	*Cricetulus barabensis*	94.65	49.45	Mongolia
289	*Cricetulus barabensis*	94.65	49.46	Mongolia
290	*Cricetulus barabensis*	94.64	49.44	Mongolia
291	*Cricetulus barabensis*	91.65	50.04	Mongolia
292	*Cricetulus barabensis*	91.65	50.04	Mongolia
293	*Cricetulus barabensis*	91.64	50.03	Mongolia
294	*Cricetulus barabensis*	94.65	49.45	Mongolia
295	*Cricetulus barabensis*	91.65	50.04	Mongolia
296	*Cricetulus barabensis*	91.64	50.03	Mongolia
297	*Cricetulus barabensis*	94.65	49.46	Mongolia
298	*Cricetulus barabensis*	94.65	49.44	Mongolia
299	*Cricetulus barabensis*	94.65	49.45	Mongolia
300	*Cricetulus barabensis*	94.63	49.44	Mongolia
301	*Cricetulus barabensis*	94.65	49.45	Mongolia
302	*Cricetulus barabensis*	94.65	49.45	Mongolia
303	*Cricetulus barabensis*	94.65	49.45	Mongolia
304	*Cricetulus barabensis*	94.65	49.45	Mongolia
305	*Cricetulus barabensis*	91.65	50.04	Mongolia

306	*Cricetulus barabensis*	94.65	49.44	Mongolia
307	*Cricetulus barabensis*	94.64	49.44	Mongolia
308	*Cricetulus barabensis*	91.19	49.87	Mongolia
309	*Cricetulus barabensis*	91.21	49.87	Mongolia
310	*Cricetulus barabensis*	91.19	49.87	Mongolia
311	*Cricetulus barabensis*	91.21	49.87	Mongolia
312	*Cricetulus barabensis*	91.20	49.87	Mongolia
313	*Cricetulus barabensis*	91.21	49.87	Mongolia
314	*Cricetulus barabensis*	91.20	49.87	Mongolia
315	*Cricetulus barabensis*	100.55	50.54	Mongolia
316	*Cricetulus barabensis*	100.55	50.54	Mongolia
317	*Cricetulus barabensis*	100.53	50.54	Mongolia
318	*Cricetulus barabensis*	100.53	50.54	Mongolia
319	*Cricetulus barabensis*	100.53	50.54	Mongolia
320	*Cricetulus barabensis*	94.65	49.46	Mongolia
321	*Cricetulus barabensis*	94.65	49.46	Mongolia
322	*Cricetulus barabensis*	100.55	50.54	Mongolia
323	*Cricetulus barabensis*	100.53	50.54	Mongolia
324	*Cricetulus barabensis*	91.20	49.87	Mongolia
325	*Cricetulus barabensis*	94.65	49.46	Mongolia
326	*Cricetulus barabensis*	94.65	49.46	Mongolia
327	*Cricetulus barabensis*	94.66	49.46	Mongolia
328	*Cricetulus barabensis*	94.65	49.46	Mongolia
329	*Cricetulus barabensis*	94.63	49.44	Mongolia
330	*Cricetulus barabensis*	94.63	49.44	Mongolia
331	*Cricetulus barabensis*	94.62	49.44	Mongolia
332	*Cricetulus barabensis*	94.62	49.44	Mongolia
333	*Cricetulus barabensis*	91.21	49.87	Mongolia
334	*Cricetulus barabensis*	94.65	49.45	Mongolia
335	*Cricetulus barabensis*	100.16	46.86	Mongolia
336	*Cricetulus barabensis*	100.15	46.86	Mongolia
337	*Cricetulus barabensis*	100.16	46.86	Mongolia
338	*Cricetulus barabensis*	100.14	46.89	Mongolia
339	*Cricetulus barabensis*	107.13	47.75	Mongolia
340	*Cricetulus barabensis*	106.55	48.26	Mongolia
341	*Cricetulus barabensis*	105.24	47.87	Mongolia
342	*Cricetulus barabensis*	101.96	46.79	Mongolia
343	*Cricetulus barabensis*	107.38	47.88	Mongolia
344	*Cricetulus barabensis*	107.33	47.82	Mongolia
345	*Cricetulus barabensis*	104.97	37.50	China
346	*Cricetulus barabensis*	93.60	43.32	China
347	*Cricetulus barabensis*	87.58	43.80	China
348	*Cricetulus barabensis*	93.45	42.47	China
349	*Cricetulus barabensis*	103.75	47.55	Mongolia

350	*Cricetulus barabensis*	103.75	47.64	Mongolia
351	*Cricetulus barabensis*	93.42	51.05	Russia
352	*Cricetulus barabensis*	94.97	50.07	Russia
353	*Cricetulus barabensis*	-91.18	30.41	United States of America
354	*Cricetulus barabensis*	103.88	46.95	Mongolia
355	*Cricetulus barabensis*	117.74	48.96	China
356	*Cricetulus barabensis*	122.25	48.08	China
357	*Cricetulus barabensis*	122.12	48.55	China
358	*Cricetulus barabensis*	122.72	48.17	China
359	*Cricetulus barabensis*	126.65	45.75	China
360	*Cricetulus barabensis*	126.65	45.75	China
361	*Cricetulus barabensis*	75.68	55.18	Russia
362	*Cricetulus barabensis*	117.70	52.23	Russia
363	*Cricetulus barabensis*	117.70	52.23	Russia
364	*Cricetulus barabensis*	101.47	45.15	Mongolia
365	*Cricetulus barabensis*	106.88	50.32	Russia
366	*Cricetulus barabensis*	119.73	40.02	China
367	*Cricetulus barabensis*	102.33	46.75	Mongolia
368	*Cricetulus barabensis*	101.47	45.15	Mongolia
369	*Cricetulus barabensis*	111.65	40.81	China
370	*Cricetulus barabensis*	119.17	25.83	China
371	*Cricetulus barabensis*	111.65	40.82	China
372	*Cricetulus barabensis*	77.88	53.72	Russia
373	*Cricetulus barabensis*	99.90	48.17	Mongolia
374	*Cricetulus barabensis*	105.50	50.58	Russia
375	*Cricetulus barabensis*	103.65	47.38	Mongolia
376	*Cricetulus barabensis*	119.70	49.20	China
377	*Cricetulus barabensis*	60.00	60.00	Russia
378	*Cricetulus barabensis*	78.16	53.53	Kazakhstan
379	*Cricetulus barabensis*	78.04	53.73	Russia
380	*Cricetulus barabensis*	80.48	54.33	Russia
381	*Cricetulus barabensis*	81.03	52.90	Russia
382	*Cricetulus barabensis*	82.22	52.62	Russia
383	*Cricetulus barabensis*	84.68	51.40	Russia
384	*Cricetulus barabensis*	87.93	50.23	Russia
385	*Cricetulus barabensis*	90.67	50.27	Russia
386	*Cricetulus barabensis*	91.79	50.54	Mongolia
387	*Cricetulus barabensis*	91.95	49.68	Mongolia
388	*Cricetulus barabensis*	95.12	50.05	Russia
389	*Cricetulus barabensis*	94.91	50.27	Russia
390	*Cricetulus barabensis*	95.07	50.33	Russia
391	*Cricetulus barabensis*	94.95	50.62	Russia
392	*Cricetulus barabensis*	94.70	51.00	Russia
393	*Cricetulus barabensis*	94.94	51.29	Russia

394	*Cricetulus barabensis*	94.63	51.60	Russia
395	*Cricetulus barabensis*	94.38	52.10	Russia
396	*Cricetulus barabensis*	95.70	51.37	Russia
397	*Cricetulus barabensis*	99.70	49.66	Mongolia
398	*Cricetulus barabensis*	101.05	50.46	Mongolia
399	*Cricetulus barabensis*	101.74	50.47	Mongolia
400	*Cricetulus barabensis*	97.30	48.66	Mongolia
401	*Cricetulus barabensis*	99.38	48.63	Mongolia
402	*Cricetulus barabensis*	99.39	48.27	Mongolia
403	*Cricetulus barabensis*	99.45	48.33	Mongolia
404	*Cricetulus barabensis*	99.65	48.16	Mongolia
405	*Cricetulus barabensis*	99.90	48.17	Mongolia
406	*Cricetulus barabensis*	100.39	48.02	Mongolia
407	*Cricetulus barabensis*	101.84	47.35	Mongolia
408	*Cricetulus barabensis*	102.97	46.75	Mongolia
409	*Cricetulus barabensis*	102.66	47.03	Mongolia
410	*Cricetulus barabensis*	102.89	47.24	Mongolia
411	*Cricetulus barabensis*	105.97	50.58	Russia
412	*Cricetulus barabensis*	105.50	50.58	Russia
413	*Cricetulus barabensis*	106.10	50.60	Russia
414	*Cricetulus barabensis*	106.68	51.00	Russia
415	*Cricetulus barabensis*	106.67	50.93	Russia
416	*Cricetulus barabensis*	107.46	51.97	Russia
417	*Cricetulus barabensis*	111.97	49.60	Russia
418	*Cricetulus barabensis*	106.82	53.10	Russia
419	*Cricetulus barabensis*	112.72	52.09	Russia
420	*Cricetulus barabensis*	113.56	52.00	Russia
421	*Cricetulus barabensis*	113.59	51.01	Russia
422	*Cricetulus barabensis*	114.29	51.08	Russia
423	*Cricetulus barabensis*	115.03	50.53	Russia
424	*Cricetulus barabensis*	115.59	52.19	Russia
425	*Cricetulus barabensis*	116.53	51.98	Russia
426	*Cricetulus barabensis*	116.92	51.71	Russia
427	*Cricetulus barabensis*	117.22	51.77	Russia
428	*Cricetulus barabensis*	117.12	50.94	Russia
429	*Cricetulus barabensis*	117.71	50.86	Russia
430	*Cricetulus barabensis*	118.32	51.10	Russia
431	*Cricetulus barabensis*	117.94	50.92	Russia
432	*Cricetulus barabensis*	118.34	51.55	Russia
433	*Cricetulus barabensis*	128.78	51.59	Russia
434	*Cricetulus barabensis*	128.25	50.14	Russia
435	*Cricetulus barabensis*	128.87	49.99	Russia
436	*Cricetulus barabensis*	130.09	49.42	Russia
437	*Cricetulus barabensis*	131.09	47.69	Russia

438	*Cricetulus barabensis*	132.62	47.94	Russia
439	*Cricetulus barabensis*	132.77	44.75	Russia
440	*Cricetulus barabensis*	131.55	44.69	Russia
441	*Cricetulus barabensis*	131.85	44.33	Russia
442	*Cricetulus barabensis*	103.00	48.70	Mongolia
443	*Cricetulus barabensis*	103.65	48.55	Mongolia
444	*Cricetulus barabensis*	103.33	47.57	Mongolia
445	*Cricetulus barabensis*	103.65	47.38	Mongolia
446	*Cricetulus barabensis*	104.20	47.18	Mongolia
447	*Cricetulus barabensis*	104.49	47.69	Mongolia
448	*Cricetulus barabensis*	105.05	47.60	Mongolia
449	*Cricetulus barabensis*	105.66	47.22	Mongolia
450	*Cricetulus barabensis*	105.45	48.03	Mongolia
451	*Cricetulus barabensis*	105.13	48.42	Mongolia
452	*Cricetulus barabensis*	105.34	49.15	Mongolia
453	*Cricetulus barabensis*	106.05	50.00	Mongolia
454	*Cricetulus barabensis*	106.07	50.14	Mongolia
455	*Cricetulus barabensis*	106.26	50.13	Russia
456	*Cricetulus barabensis*	106.27	50.37	Russia
457	*Cricetulus barabensis*	106.73	50.33	Russia
458	*Cricetulus barabensis*	106.50	50.78	Russia
459	*Cricetulus barabensis*	106.58	50.87	Russia
460	*Cricetulus barabensis*	106.62	50.97	Russia
461	*Cricetulus barabensis*	115.97	51.27	Russia
462	*Cricetulus barabensis*	116.23	51.42	Russia
463	*Cricetulus barabensis*	114.82	50.49	Russia
464	*Cricetulus barabensis*	115.13	50.51	Russia
465	*Cricetulus barabensis*	115.34	50.10	Russia
466	*Cricetulus barabensis*	115.70	50.00	Russia
467	*Cricetulus barabensis*	115.68	50.10	Russia
468	*Cricetulus barabensis*	116.18	50.55	Russia
469	*Cricetulus barabensis*	117.16	50.47	Russia
470	*Cricetulus barabensis*	118.03	50.09	Russia
471	*Cricetulus barabensis*	117.60	50.37	Russia
472	*Cricetulus barabensis*	118.11	50.26	Russia
473	*Cricetulus barabensis*	118.40	50.42	Russia
474	*Cricetulus barabensis*	118.67	50.33	Russia
475	*Cricetulus barabensis*	118.93	50.01	Russia
476	*Cricetulus barabensis*	119.27	50.61	Russia
477	*Cricetulus barabensis*	118.93	51.21	Russia
478	*Cricetulus barabensis*	119.50	51.22	Russia
479	*Cricetulus barabensis*	119.61	51.31	Russia
480	*Cricetulus barabensis*	119.61	46.98	Mongolia
481	*Cricetulus barabensis*	112.19	48.86	Mongolia

482	*Cricetulus barabensis*	111.94	48.61	Mongolia
483	*Cricetulus barabensis*	111.71	48.29	Mongolia
484	*Cricetulus barabensis*	116.06	43.94	China
485	*Cricetulus barabensis*	116.92	40.50	China
486	*Cricetulus barabensis*	114.85	34.05	Mongolia
487	*Cricetulus barabensis*	107.61	37.61	China
488	*Cricetulus barabensis*	107.61	37.57	China
489	*Cricetulus barabensis*	107.88	37.73	China
490	*Cricetulus barabensis*	108.86	40.86	China
491	*Cricetulus barabensis*	109.37	32.77	China
492	*Cricetulus barabensis*	109.96	39.06	China
493	*Cricetulus barabensis*	110.00	38.64	China
494	*Cricetulus barabensis*	111.83	40.86	China
495	*Cricetulus barabensis*	112.32	32.41	China
496	*Cricetulus barabensis*	112.38	32.52	China
497	*Cricetulus barabensis*	112.39	32.73	China
498	*Cricetulus barabensis*	112.56	32.60	China
499	*Cricetulus barabensis*	113.31	36.26	China
500	*Cricetulus barabensis*	114.05	34.85	China
501	*Cricetulus barabensis*	114.17	44.63	China
502	*Cricetulus barabensis*	114.74	45.00	China
503	*Cricetulus barabensis*	114.94	41.39	China
504	*Cricetulus barabensis*	115.16	42.63	China
505	*Cricetulus barabensis*	115.80	44.77	China
506	*Cricetulus barabensis*	116.20	45.01	China
507	*Cricetulus barabensis*	116.30	34.54	China
508	*Cricetulus barabensis*	116.41	39.63	China
509	*Cricetulus barabensis*	116.59	39.81	China
510	*Cricetulus barabensis*	116.72	40.14	China
511	*Cricetulus barabensis*	116.73	39.95	China
512	*Cricetulus barabensis*	116.98	35.60	China
513	*Cricetulus barabensis*	117.00	35.63	China
514	*Cricetulus barabensis*	117.01	35.78	China
515	*Cricetulus barabensis*	117.09	39.39	China
516	*Cricetulus barabensis*	118.65	34.91	China
517	*Cricetulus barabensis*	118.70	35.50	China
518	*Cricetulus barabensis*	118.70	36.50	China
519	*Cricetulus barabensis*	119.39	35.35	China
520	*Cricetulus barabensis*	119.64	29.12	China
521	*Cricetulus barabensis*	119.68	30.68	China
522	*Cricetulus barabensis*	120.32	28.84	China
523	*Cricetulus barabensis*	120.70	36.91	China
524	*Cricetulus barabensis*	120.72	37.03	China
525	*Cricetulus barabensis*	121.03	29.15	China

526	*Cricetulus barabensis*	124.10	42.78	China
527	*Cricetulus barabensis*	124.91	43.77	China
528	*Cricetulus barabensis*	125.70	44.00	China
529	*Cricetulus barabensis*	125.90	43.40	China
530	*Cricetulus barabensis*	126.31	43.24	China
531	*Cricetulus barabensis*	126.60	47.45	China
532	*Cricetulus barabensis*	126.82	45.86	China
533	*Cricetulus barabensis*	126.96	47.46	China
534	*Cricetulus barabensis*	127.95	43.49	China
535	*Cricetulus barabensis*	128.00	43.92	China
536	*Cricetulus barabensis*	117.00	35.77	China
537	*Cricetulus barabensis*	117.00	35.77	China
538	*Cricetulus barabensis*	115.67	38.12	China
539	*Cricetulus barabensis*	116.00	44.97	China
540	*Cricetulus barabensis*	115.22	38.20	China
541	*Cricetulus barabensis*	111.75	40.60	China
542	*Cricetulus barabensis*	111.11	40.85	China
543	*Cricetulus barabensis*	117.89	48.62	China
544	*Cricetulus barabensis*	115.50	44.70	China
545	*Cricetulus barabensis*	127.02	46.65	China
546	*Cricetulus barabensis*	124.67	46.82	China
547	*Cricetulus barabensis*	117.25	39.42	China
548	*Cricetulus barabensis*	118.00	48.00	China
549	*Cricetulus barabensis*	104.08	38.66	China
550	*Cricetulus barabensis*	104.83	37.90	China
551	*Cricetulus barabensis*	115.99	43.64	China
552	*Cricetulus barabensis*	111.57	38.70	China
553	*Cricetulus barabensis*	118.12	36.92	China
554	*Cricetulus barabensis*	116.05	43.12	China
555	*Cricetulus barabensis*	120.34	44.21	China
556	*Cricetulus barabensis*	122.73	46.00	China
557	*Cricetulus barabensis*	123.93	47.37	China
558	*Cricetulus barabensis*	124.12	42.78	China
559	*Cricetulus barabensis*	115.43	39.97	China
560	*Cricetulus barabensis*	110.58	40.70	China
561	*Cricetulus barabensis*	117.45	38.83	China
562	*Cricetulus barabensis*	122.05	46.22	China

Supplementary Table 2. Primers used in this study for qPCR

Gene	Forward (5’→3’)	Reverse (5’→3’)
CD11b	AAGCTCTTCTGGTCACAGCC	AAGCTCTTCTGGTCACAGCC
CD14	ACTGAAGCCTTTCTCGGAGC	AAGCACACGCTCCATGGTC
CD68	TTACCTTTGGATTCAAACAGGACC	GAGGCAGCAAGAGGGACTG

CX3CR1	ACCGGTACCTTGCCATCGT	ACACCGTGCTGCACTGTCC
DAP12	GAGTGACACTTTCCCAAGATG	CCTTGACCTCGGGAGACC
FCER1G	ATCTCAGCCTGTATCTTGTTCT	ACCATACAAAAACAGGACAGCAT
FCGR2	ATCTTGCTGCTGGGACTCAT	TGACTGTGGCCTTAAACGTG
RAC2	GACAGTAAGCCGGTGAACCTG	CTGACTAGCGAGAAGCAGATG
TMEM119	TCACCCAGAGCTGGTTCCAT	GTGACACAGAGTAGGCCACC
CLAUDIN1	CTGGGATGGATCGGCTCTATC	CCTCGTAGATGGCCTGAGCA
OCCU1	TGACATGTATGGCGGAGAGATG	CCTCGTAGATGGCCTGAGCA
TJP1	CCAGCAACTTTCAGACCACC	TTGTGTACGGCTTTGGTGTG
GAPDH	GGCAAATTCAACGGCACAGT	AGATGGTGATGGGCTTCCC
