# Supplementary material for: Ectoparasites enhance survival by suppressing host exploration and limiting dispersal
Source: Nat Commun. 2025 May 9;16:4318. doi: 10.1038/s41467-025-59601-9 (PMC12064801; doi:10.1038/s41467-025-59601-9)
Supplement: Supplementary file 2 — Description Of Additional Supplementary File [file 41467_2025_59601_MOESM2_ESM.pdf]

Description of Additional supplementary files

Title: Supplementary data 1 - PFC

Description: The differentially expressed genes in the prefrontal cortex (PFC) of Flea+ mice compared to Flea- based on transcriptomic data

Title: Supplementary data 1 - HC

Description: Differentially expressed genes in the hippocampal (HC) of Flea+ vs. Flea- mice based on transcriptomic data

Title: Supplementary data 1 - TH

Description: Differentially expressed genes in the thalamus (TH) of Flea+ vs. Flea- mice based on transcriptomic data

Title: Supplementary data 2 – PFC_up

Description: GO enrichment analysis of upregulated differentially expressed genes in the prefrontal cortex (PFC) of Flea+ mice compared to Flea- group

Title: Supplementary data 2 – PFC_down

Description: GO enrichment analysis of downregulated differentially expressed genes in the prefrontal cortex (PFC) of Flea+ mice relative to Flea- group

Title: Supplementary data 2 – HC_up

Description: GO enrichment analysis of upregulated differentially expressed genes in the hippocampal (HC) of Flea+ mice compared to Flea- group

Title: Supplementary data 2 – HC_down

Description: GO enrichment analysis of downregulated differentially expressed genes in the hippocampal (HC) of Flea+ mice relative to Flea- group

Title: Supplementary data 2 – TH_up

Description: GO enrichment analysis of upregulated differentially expressed genes in the thalamus (TH) of Flea+ mice compared to Flea- group

Title: Supplementary data 2 – TH_down

Description: GO enrichment analysis of downregulated differentially expressed genes in the thalamus (TH) of Flea+ mice relative to Flea- group
