# Supplementary material for: Ectoparasites enhance survival by suppressing host exploration and limiting dispersal
Source: Nat Commun. 2025 May 9;16:4318. doi: 10.1038/s41467-025-59601-9 (PMC12064801; doi:10.1038/s41467-025-59601-9)
Supplement: Supplementary file 5 — Reporting summary [file 41467_2025_59601_MOESM5_ESM.pdf]

nature portfolio

Corresponding author(s): Liang Lu

Last updated by author(s): Apr 11, 2025

Reporting Summary

Nature Portfolio wishes to improve the reproducibility of the work that we publish. This form provides structure for consistency and transparency in reporting. For further information on Nature Portfolio policies, see our Editorial Policies and the Editorial Policy Checklist.

Statistics

For all statistical analyses, confirm that the following items are present in the figure legend, table legend, main text, or Methods section.

n/a	Confirmed	
☐	☒	The exact sample size (*n*) for each experimental group/condition, given as a discrete number and unit of measurement
☐	☒	A statement on whether measurements were taken from distinct samples or whether the same sample was measured repeatedly
☐	☒	The statistical test(s) used AND whether they are one- or two-sided<br>*Only common tests should be described solely by name; describe more complex techniques in the Methods section.*
☒	☐	A description of all covariates tested
☐	☒	A description of any assumptions or corrections, such as tests of normality and adjustment for multiple comparisons
☐	☒	A full description of the statistical parameters including central tendency (e.g. means) or other basic estimates (e.g. regression coefficient) AND variation (e.g. standard deviation) or associated estimates of uncertainty (e.g. confidence intervals)
☒	☐	For null hypothesis testing, the test statistic (e.g. *F*, *t*, *r*) with confidence intervals, effect sizes, degrees of freedom and *P* value noted<br>*Give P values as exact values whenever suitable.*
☒	☐	For Bayesian analysis, information on the choice of priors and Markov chain Monte Carlo settings
☒	☐	For hierarchical and complex designs, identification of the appropriate level for tests and full reporting of outcomes
☒	☐	Estimates of effect sizes (e.g. Cohen's *d*, Pearson's *r*), indicating how they were calculated

Our web collection on statistics for biologists contains articles on many of the points above.

Software and code

Policy information about availability of computer code

Data collection	*Provide a description of all commercial, open source and custom code used to collect the data in this study, specifying the version used OR state that no software was used.*
Data analysis	*Provide a description of all commercial, open source and custom code used to analyse the data in this study, specifying the version used OR state that no software was used.*

For manuscripts utilizing custom algorithms or software that are central to the research but not yet described in published literature, software must be made available to editors and reviewers. We strongly encourage code deposition in a community repository (e.g. GitHub). See the Nature Portfolio guidelines for submitting code & software for further information.

Data

Policy information about availability of data

All manuscripts must include a data availability statement. This statement should provide the following information, where applicable:

- Accession codes, unique identifiers, or web links for publicly available datasets
- A description of any restrictions on data availability
- For clinical datasets or third party data, please ensure that the statement adheres to our policy

The raw RNA-seq data generated in this study have been deposited in Dryad under accession code [DOI: 10.5061/dryad.x3ffbg7vh]. The raw neurotransmitter data are available at Dryad under accession code [DOI: 10.5061/dryad.7d7wm384n]. Source Data are provided with this paper.

Research involving human participants, their data, or biological material

Policy information about studies with human participants or human data. See also policy information about sex, gender (identity/presentation), and sexual orientation and race, ethnicity and racism.

Reporting on sex and gender	*Use the terms sex (biological attribute) and gender (shaped by social and cultural circumstances) carefully in order to avoid confusing both terms. Indicate if findings apply to only one sex or gender; describe whether sex and gender were considered in study design; whether sex and/or gender was determined based on self-reporting or assigned and methods used. Provide in the source data disaggregated sex and gender data, where this information has been collected, and if consent has been obtained for sharing of individual-level data; provide overall numbers in this Reporting Summary. Please state if this information has not been collected. Report sex- and gender-based analyses where performed, justify reasons for lack of sex- and gender-based analysis.*
Reporting on race, ethnicity, or other socially relevant groupings	*Please specify the socially constructed or socially relevant categorization variable(s) used in your manuscript and explain why they were used. Please note that such variables should not be used as proxies for other socially constructed/relevant variables (for example, race or ethnicity should not be used as a proxy for socioeconomic status). Provide clear definitions of the relevant terms used, how they were provided (by the participants/respondents, the researchers, or third parties), and the method(s) used to classify people into the different categories (e.g. self-report, census or administrative data, social media data, etc.) Please provide details about how you controlled for confounding variables in your analyses.*
Population characteristics	*Describe the covariate-relevant population characteristics of the human research participants (e.g. age, genotypic information, past and current diagnosis and treatment categories). If you filled out the behavioural & social sciences study design questions and have nothing to add here, write "See above."*
Recruitment	*Describe how participants were recruited. Outline any potential self-selection bias or other biases that may be present and how these are likely to impact results.*
Ethics oversight	*Identify the organization(s) that approved the study protocol.*

Note that full information on the approval of the study protocol must also be provided in the manuscript.

Field-specific reporting

Please select the one below that is the best fit for your research. If you are not sure, read the appropriate sections before making your selection.

☐ Life sciences ☐ Behavioural & social sciences ☒ Ecological, evolutionary & environmental sciences

For a reference copy of the document with all sections, see nature.com/documents/nr-reporting-summary-flat.pdf

Ecological, evolutionary & environmental sciences study design

All studies must disclose on these points even when the disclosure is negative.

Study description	We conducted behavioral tests on laboratory mice following flea infection to validate the hypothesis that ectoparasites reduce host exploratory behavior, and explored the underlying neuro-molecular mechanisms. To extrapolate the findings, we performed indoor infection and enclosure experiments with wild-caught striped hamsters. Additionally, we used SDM and RangeshiftR models to simulate the phenomenon of flea bites reducing host exploratory behavior at a macroecological scale.
Research sample	3-week-old female C57BL/6 mice, healthy, housed in controlled laboratory conditions;<br>Wild-caught striped hamsters;
Sampling strategy	For the laboratory mice, we selected over 100 healthy 3-week-old female C57BL/6 mice to ensure robust data and meet the requirements for various metrics. These mice were acclimated to laboratory conditions and randomly assigned to the Flea+ or Flea- groups. For the wild black-tailed hamsters, we ensured the minimum required sample size per group to meet statistical testing standards.
Data collection	We trained other laboratory staff to ensure consistency in data collection. Flea+ and Flea- mice were randomly selected from their cages for behavioral experiments. Data analysis was conducted following standardized protocols to ensure uniformity and accuracy in the evaluation of behavioral outcomes.<br>In this study, data on striped hamsters were sourced from three areas: our team's field sampling records, literature reports, and the Global Biodiversity Information Facility (GBIF, https://www.gbif.org). We sourced climate data from the WorldClim database (http://worldclim.org/)
Timing and spatial scale	average monthly climate data, including minimum, mean, and maximum temperature, as well as precipitation for the period 1970–2000, with a spatial resolution of 5 arcminutes;<br>climate variables under four Shared Socioeconomic Pathways (SSP126, SSP245, SSP370, SSP585) for three future time periods (2041~2060, 2061~2080, 2081~2100)

Data exclusions	After compiling data from these three sources, we cleaned the dataset by removing duplicates, points with zero coordinates, points located at sea, capital cities, and institutional coordinates. We retained only unique distribution points within a 5 km radius
Reproducibility	For omics data and PET/CT imaging, we ensured that each group had at least 3 biological replicates. For all other tests, we ensured a minimum of 5 biological replicates per group, guaranteeing the reproducibility and robustness of our results.
Randomization	Randomization was applied to assign them to either the Flea+ or Control group, ensuring that each group was statistically comparable and minimizing selection bias.
Blinding	In the behavioral tests, the experimenters were blinded to the group allocation of the mice.

Did the study involve field work? ☒ Yes ☐ No

Field work, collection and transport

Field conditions	The region spans a vast expanse of 2400 km from east to west and exhibits a precipitation gradient difference of 300 mm per annum, as well as a maximum north-south distance of 1700 km, with an annual temperature difference exceeding 10?.
Location	in the grasslands of Inner Mongolia Autonomous Region, China
Access & import/export	We collaborated with the local Centers for Disease Control (CDC) for academic support, covering everything from field sampling to the construction of the enclosures. The dissected tissues were frozen in liquid nitrogen and transported to Beijing via vehicle, ensuring that no contact with unrelated personnel occurred throughout the entire process. This approach maintained the integrity and security of the biological samples.
Disturbance	We took measures to minimize any disturbance to the animals during the study. Hamsters were handled gently during the behavioral tests and sampling procedures. The field trapping and enclosure experiments were conducted with minimal interference to the natural environment, and the handling of tissues was carried out under controlled conditions to avoid any potential disruption to the samples.

Reporting for specific materials, systems and methods

We require information from authors about some types of materials, experimental systems and methods used in many studies. Here, indicate whether each material, system or method listed is relevant to your study. If you are not sure if a list item applies to your research, read the appropriate section before selecting a response.

Materials & experimental systems

n/a	Involved in the study	
☐	☒	Antibodies
☒	☐	Eukaryotic cell lines
☒	☐	Palaeontology and archaeology
☐	☒	Animals and other organisms
☒	☐	Clinical data
☒	☐	Dual use research of concern
☐	☒	Plants

Methods

n/a	Involved in the study	
☒	☐	ChIP-seq
☐	☒	Flow cytometry
☒	☐	MRI-based neuroimaging

Antibodies

Antibodies used	rabbitanti-IBA1 (1:500, 10904-1-AP, Proteintech), mouse anti-NeuN (1:100, ab104224, Abcam), mouse anti-TUNEL (1:100, A112-03, Vazyme), rabbit anti-PSD95 (1:100, ab238135, Abcam), rabbitanti-HOMER1 (1:100, A4302, Abbiotec), mouse anti-Synaptophysin (1:100, ab8049, Abcam), rabbit anti-GAD65/67 (1:100, ab183999, Abcam), rabbitanti-GABRG2 (1:100, 14104-1-AP, Proteintech), and rabbitanti-VGLUT1 (1:100, 55491-1-AP, Proteintech); goat anti-mouse IgG (1:100, BA1031, BOSTER) ;goat anti-rabbit IgG (1:100, BA1032, BOSTER).
Validation	We confirm that we used these antibodies according to the manufacturer's instructions and guidelines. All procedures were performed following the recommended protocols to ensure accuracy and reliability.

Animals and other research organisms

Policy information about studies involving animals; ARRIVE guidelines recommended for reporting animal research, and Sex and Gender in Research

Laboratory animals	C57BL/6 mice

Wild animals	We conducted an infection experiment with striped hamsters in Xilinhot, Inner Mongolia, China. Striped hamsters, a dominant species in the Xilingol grassland, live solitarily, making their ectoparasite load a reliable indicator of individual infection levels. The hamsters were live-trapping using Sherman traps in West Ujimqin Banner . Each morning, the traps were checked, and the captured hamsters were brought back to the laboratory for weighing. Fleas were combed from their fur to establish experimental populations.
Reporting on sex	We only used female C57BL/6 mice in our laboratory experiments, while both male and female wild black-tailed hamsters were included in our field study.
Field-collected samples	The hamsters were housed individually in cages under natural light conditions. To eliminate interference from wild-caught fleas, the hamsters were kept in cages for at least four weeks before being used in the infection experiment.
Ethics oversight	This study received ethical approval from the Ethical Committee of the National Institute for Communicable Disease Control and Prevention, Chinese Center for Disease Control and Prevention(No.2022-027).

Note that full information on the approval of the study protocol must also be provided in the manuscript.

Dual use research of concern

Policy information about dual use research of concern

Hazards

Could the accidental, deliberate or reckless misuse of agents or technologies generated in the work, or the application of information presented in the manuscript, pose a threat to:

No	Yes	
☒	☐	Public health
☒	☐	National security
☒	☐	Crops and/or livestock
☒	☐	Ecosystems
☒	☐	Any other significant area

Experiments of concern

Does the work involve any of these experiments of concern:

No	Yes	
☒	☐	Demonstrate how to render a vaccine ineffective
☒	☐	Confer resistance to therapeutically useful antibiotics or antiviral agents
☒	☐	Enhance the virulence of a pathogen or render a nonpathogen virulent
☒	☐	Increase transmissibility of a pathogen
☒	☐	Alter the host range of a pathogen
☒	☐	Enable evasion of diagnostic/detection modalities
☒	☐	Enable the weaponization of a biological agent or toxin
☒	☐	Any other potentially harmful combination of experiments and agents

Plants

Seed stocks	We assessed plant diversity within the enclosures by surveying plant cover, biomass, and density in June, 2021. In each enclosure, four 1m x 1m quadrats were randomly selected to measure the cover percentage, density (number of rooted plants per quadrat), and biomass (g/m²) of each plant species. Each 1m x 1m quadrat was further divided into 16 sub-quadrats (0.25m x 0.25m), and in one sub-quadrat, the above-ground parts of each plant species were clipped at ground level.
Novel plant genotypes	Not applicable
Authentication	Not applicable

Flow Cytometry

Plots

Confirm that:

☒ The axis labels state the marker and fluorochrome used (e.g. CD4-FITC).

☒ The axis scales are clearly visible. Include numbers along axes only for bottom left plot of group (a 'group' is an analysis of identical markers).

☐ All plots are contour plots with outliers or pseudocolor plots.

☒ A numerical value for number of cells or percentage (with statistics) is provided.

Methodology

Sample preparation	The PFC was carefully rinsed with PBS and then immersed in PBS containing 5% FBS. To achieve red blood cell lysis, the tissue was incubated with 10 ml of 0.25% Trypsin-EDTA at 37°C for 10 minutes, with gentle mixing and pipetting to dissociate the cells. The cell suspension was filtered through a Falcon® 70 µm Cell Strainer, and any remaining tissue was gently ground on the strainer and rinsed with RPMI 1640 containing 5% FBS. The filtration was repeated twice, followed by centrifugation at 300g for 5 minutes. The supernatant was discarded, and the pellet was re-suspended in sterile PBS to achieve a cell concentration of $2x10^7$ cells/ml, then stored at 4°C until further use.
Instrument	Cytek NL-CLC3000 flow cytometer
Software	SpectroFlo 1.0 software
Cell population abundance	For each sample, 100 µl of the cell suspension (approximately $2x10^6$ cells) was transferred to the bottom of a flow cytometry tube.
Gating strategy	In flow cytometry analysis, we used CD11b and CD45 markers to identify microglia in the prefrontal cortex of mice, with the following gating strategy: First, singlet cells were selected by gating on forward scatter (FSC) and side scatter (SSC) parameters to remove aggregates and doublets. Next, live cells were selected using a viability dye (such as 7-AAD or PI) to exclude dead cells. Then, microglia were isolated based on CD11b and CD45 expression, with microglia typically being CD11b + and CD45low. Finally, microglia were further gated in the P4 region for subsequent analysis.

☒ Tick this box to confirm that a figure exemplifying the gating strategy is provided in the Supplementary Information.
